# Time trends, geographical, socio-economic, and gender disparities in neonatal mortality in Burundi: evidence from the demographic and health surveys, 2010–2016

**DOI:** 10.1186/s13690-020-00501-3

**Published:** 2020-11-12

**Authors:** Sanni Yaya, Betregiorgis Zegeye, Bright Opoku Ahinkorah, Edward Kwabena Ameyaw, Abdul-Aziz Seidu, Gebretsadik Shibre

**Affiliations:** 1 School of International Development and Global Studies, University of Ottawa, Ottawa, Canada; 2 The George Institute for Global Health, Imperial College London, London, UK; 3 Shewarobit Field Office, HaSET Maternal and Child Health Research Program, Addis Ababa, Ethiopia; 4 School of Public Health, Faculty of Health, University of Technology Sydney, Ultimo, NSW Australia; 5 Department of Population and Health, College of Humanities and Legal Studies, University of Cape Coast, Cape Coast, Ghana; 6 Department of Reproductive, Family and Population Health, School of Public Health, Addis Ababa University, Addis Ababa, Ethiopia

**Keywords:** Neonatal mortality, Magnitude, Time trends, Inequality, Global health, Burundi, DHS

## Abstract

**Background:**

Programmatic and research agendas surrounding neonatal mortality are important to help countries attain the child health related 2030 Sustainable Development Goal (SDG). In Burundi, the Neonatal Mortality Rate (NMR) is 25 per 1000 live births. However, high quality evidence on the over time evolution of inequality in NMR is lacking. This study aims to address the knowledge gap by systematically and comprehensively investigating inequalities in NMR in Burundi with the intent to help the country attain SDG 3.2 which aims to reduce neonatal mortality to at least as low as 12 per 1000 live births by 2030.

**Methods:**

The Burundi Demographic and Health Survey (BDHS) data for the periods of 2010 and 2016 were used for the analyses. The analyses were carried out using the WHO’s HEAT version 3.1 software. Five equity stratifiers: economic status, education, residence, sex and subnational region were used as benchmark for measuring NMR inequality with time over 6 years. To understand inequalities from a broader perspective, absolute and relative inequality measures, namely Difference, Population Attributable Risk (PAR), Ratio, and Population Attributable Fraction (PAF) were calculated. Statistical significance was measured by computing corresponding 95% Confidence Intervals (CIs).

**Results:**

NMR in Burundi in 2010 and 2016 were 36.7 and 25.0 deaths per 1000 live births, respectively. We recorded large wealth-driven (PAR = -3.99, 95% CI; − 5.11, − 2.87, PAF = -15.95, 95% CI; − 20.42, − 11.48), education related (PAF = -6.64, 95% CI; − 13.27, − 0.02), sex based (PAR = -1.74, 95% CI; − 2.27, − 1.21, PAF = -6.97, 95% CI; − 9.09, − 4.86), urban-rural (D = 15.44, 95% CI; 7.59, 23.29, PAF = -38.78, 95% CI; − 45.24, − 32.32) and regional (PAR = -12.60, 95% CI; − 14.30, − 10.90, R = 3.05, 95% CI; 1.30, 4.80) disparity in NMR in both survey years, except that urban-rural disparity was not detected in 2016. We found both absolute and relative inequalities and significant reduction in these inequalities over time - except at the regional level, where the disparity remained constant during the study period.

**Conclusion:**

Large survival advantage remains to neonates of women who are rich, educated, residents of urban areas and some regions. Females had higher chance of surviving their 28th birthday than male neonates. More extensive work is required to battle the NMR gap between different subgroups in the country.

## Background

The first twenty-eight days of life – the neonatal period – is the most vulnerable time for a child’s survival. Children face the highest risk of dying in their first month of life, and a global rate of 18 deaths per 1000 live births was recorded in 2018 [1]. According to data provided by UNICEF, worldwide, there are approximately 7000 neonatal deaths every day, with one third dying on the first day [1]. Though, neonatal mortality rate globally declined from 37 deaths per 1000 live births in 1990 to 19 in 2016, the decline in the neonatal mortality rate between 1990 to 2016 was slower (49%) than the decline in mortality among children aged 1–59 months with 62% [2].

The burden of neonatal deaths is also unevenly distributed across regions and countries [2]. Two regions accounted for almost 80% of newborn deaths in 2016 – Southern Asia accounted for 39% of all such deaths and sub-Saharan Africa (SSA) accounted for 38% [2].

Generally, effective interventions to improve survival rates of neonates around the world continue to increase. However, implementation strategies necessary for these interventions remain a challenge [3]. In a multicounty analysis, McKinnon et al. (2014) reported socioeconomic inequity in neonatal deaths which tend to occur in disadvantaged sub-populations within a country [3]. As the global community works toward an equity-oriented international agenda to meet the United Nations’ SDG 3.2 which aims to reduce neonatal mortality to at least as low as 12 per 1000 live births by 2030 [4], it is imperative that interventions reach these sub-populations. Evidence-based research is necessary to outline the problem, who is affected most, and how the problem is changing over time. This, in turn, will inform policies and strategies and target sub-groups of populations who suffer most from high rates of NMR.

The majority of neonatal deaths tend to occur in Low-and Middle-Income Countries (LMICs) [5] disfavoring the poor, illiterate and rural communities [3, 6]. Neonates born to women in higher socioeconomic positions have better survival rates [5]. Although socioeconomic inequality in NMR trends seems to have decreased over the last two decades in LMICs, there remains an inequality in access to appropriate NMR interventions (i.e. skilled birth attendance), with increased survival for neonates born into wealthier households with a higher level of education [3]. According to the WHO, despite increases in the availability and utilization of maternal and child health services, Burundi’s maternal mortality remains one of the highest in the region (three times higher than in Rwanda) and in Africa (ranked 39th of 46 countries) [7]. Child and infant mortality rates are above both the sub-Saharan African average (83 per 1000 live births) and the WHO African average (81 per 1000 live births) [7].

SSA has the highest neonatal mortality rate (NMR) in the world [8]. Burundi has a NMR of 25 deaths per 1000 live births and is one of the highest in SSA [9]. A previous facility-based study in Burundi revealed that, nearly 50% of the neonatal deaths were caused by early neonatal infection, and the remaining 50% by prematurity, fetal acute, and lung disease respectively, with variations across geographic area (urban-rural) and based on newborn’s sex [10]. More recently, the call to promote health among disadvantaged populations has been echoed through other important global initiatives, notably the Commission on Social Determinants of Health [11] and the Rio Political Declaration on Social Determinants of Health [12]. Increasingly, global initiatives are orienting towards establishing health inequality monitoring practices and tangible actions to reduce health inequalities, with a focus on accountability and results [13]. Health inequity is a normative concept, defined as the avoidable and/or unjust differences in health between population subgroups [13]. Statements about health equity involve a judgment about what is deemed to be right, fair, or acceptable in a society [13, 14]. Measuring and monitoring health inequalities is a starting point from which health equity can be evaluated [13, 14]. Evidence-based research is needed to understand trends and inequalities in NMR [13, 15], so as to inform policies and strategies and target sub-groups of populations who suffer most from high rates of NMR.

In this paper, we assess the magnitude and time-trends of socio-economic, sex, and geographic disparities in NMR using different and highly rigorous inequality measurement techniques based on the Burundi demographic health survey data. To the best of our knowledge, this is the first study on the topic that aimed at answering two major questions: 1) what is the status of NMR inequality in Burundi across different equity stratifiers; and 2) how have levels of NMR inequality in different equity stratifiers changed over time between 2010 and 2016?

## Methods

### Study setting

Burundi lies astride Eastern Africa and Central Africa [16, 17] and is the third most populous country in SSA. In 2015, the country had an estimated 435 inhabitants per km^2^ and by 2040, its booming population is expected to double [17]. With a population of 11 million, it is one of the economic-disadvantaged countries due to existing political instability and violence [17]. Burundi is the least urbanized country in SSA in spite of its high population density and as at 2014, only 12% of the population lived in urban settings [17]. Burundi trails on many human development indicators and with an average per capita consumption of only US$270 per year, the country lies at the bottom of the low-income category [17]. Hunger and malnutrition are predominant in the country despite the country’s economic dependence on agriculture. In 2014, almost 70% of Burundi’s population was found to be undernourished, over three times the then Millennium Development Goals target and 60% of children under-five were suffering from stunting [17].

Over the past two decades, there have been advances in some health indicators such as under-five and maternal mortality rates and vaccination coverage in Burundi [7]. Notwithstanding, maternal, infant, and child mortality rates lag below regional averages due to political crisis in the country, coupled with inequities in health service utilization and financial barriers to healthcare access, which are predominant in low- income and rural households [7].

### Data source

The Burundi Demographic and Health Survey (BDHS) for the periods of 2010 and 2016 were used for the analyses. Both are nationally representative household surveys. DHS is a rich source of data for several reproductive and child health care service indicators, including mortality in Burundi. The BDHS is carried out every 5 years and so far, three waves have been conducted between 2005 and 2016. Since the 2005 BDHS does not contain data on NMR, we confined our analyses to the 2010 and 2016 rounds. The survey basically covered women aged 15 to 49 years that gave births 5 years prior to the respective surveys, and men aged between 15 to 59 years and children. The response rates for the women were 96.4 and 99% for the 2010 BDHS and 2016 BDHS respectively.

The methodology of the BDHS has been clearly described in the respective DHS’s final pdf document and we refer readers to the documents for detailed information on how the surveys were conducted [9, 18]. In brief, DHS is a two-stage population-based study with the first stage being large communities or villages that encompass several households. These are normally known as Enumeration Areas (EAs). EAs are selected through Probability Proportional to Size (PPS) approach so that large EAs have higher chance of being in the sample than small EAs. The EAs were selected from the list of EAs which were prepared in the Burundi Population and Housing Census. The complete list of all the EAs in the country served as sampling frame for the first stage and list of households in the selected EAs served as sampling frame for the second stage, where households are selected from each EA and all eligible individuals within the selected households are studied. The EAs and households are the primary and secondary sampling units respectively. Samples were selected so that they were representative at the national level.

### Selection of variables

Inequality is measured for NMR, which refers to the number of deaths during the first 28 completed days of life per 1000 live births in a given year or another period. The birth histories data in the DHS has information on the birth dates and age of death of neonates. The analyses involved data on live births that took place 5 years prior to the surveys.

### Measures

Five equity stratifiers: economic status, education, residence, sex and subnational region were used as benchmark for measuring NMR inequality with time over 6 years. Wealth index, which is derived from household assets and features was used to approximate economic status. Principal Component Analysis (PCA) is used to compute wealth index in DHS and classifies it as poorest, poorer, middle, richer, and richest [19]. Educational status of the mother was grouped into no education, primary, and secondary education, while urban and rural were used to define residence. Sex was categorized as male and female, and subnational region into five regions in 2010 namely Bujumbura, North, Centre-East, West, and South and into eighteen regions in 2016 such as Bubanza, Bujumbura Rural and Bururi, just to name few.

### Statistical analysis

A two-step approach was followed in measuring inequality in NMR. The first step involved a disaggregation of NMR by five equity stratifiers, namely education, economic status, residence, subnational region and sex. This was followed by an assessment of inequality using four measures of inequality, thus Difference (D), Population Attributable Risk (PAR), Population Attributable Fraction (PAF) and Ratio (R). The D is a simple, unweighted measure of inequality that portrays the absolute inequality between two subgroups. The PAR is a complex, weighted measure of inequality that shows the potential for improvement in the national level of a health indicator that could be achieved if all subgroups had the same level of health as a reference subgroup. The PAF is a complex, weighted measure of inequality that shows the potential for improvement in the national level of a health indicator, in relative terms, that could be achieved if all subgroups had the same level of health as a reference subgroup. The R is a simple, unweighted measure of inequality that shows the relative inequality between two subgroups [20]. Whereas Difference and Ratio are simple measures, the other two are complex measures. R and PAF are relative measures, but D and PAR are absolute summary measures. The selection of summary measures is based on evidence that supports the scientific significance of using both absolute and relative measures in studies involving single health inequality [21]. This is deemed essential due to the likelihood of obtaining different and even contrasting conclusions, which can lead to bias informed decisions when using either relative and absolute inequality measures alone [21]. Further, complex measures are likely to examine inequalities over time when there is a shift in the proportion of population in each dimension of inequality [21].

The difference between complex and simple measures is that whereas the former takes into consideration size of categories of a sub-group, the later does not. Again, in situations where there is a likelihood of population shift, especially during trend analysis, complex measures are more probable to show the true change in equality over time but simple measures are easy to interpret and understand [21]. Hence, in order to provide a more comprehensive analysis, there is the need to combine both simple and complex measures, in addition to relative and absolute measures in an inequality study.

The analysis was carried out using the WHO’s HEAT version 3.1 software [20]. Detailed description of the procedures followed for calculating summary measures are available in the HEAT software technical notes [20] and in the WHO handbook on health inequality monitoring [21]. Hence, only a brief description is provided here. For education, D was calculated as NMR in “un-educated” group minus NMR in “secondary education” group, whereas for economic status, it was calculated as NMR in the poorest group minus NMR in the richest group. Similarly, D was calculated as NMR in rural minus NMR in urban populations with respect to place of residence, male minus female for sex, and region with the highest estimate minus the one with the lowest estimate in relation to subnational region. The same subgroups are used to calculate R, but instead of subtracting NMR estimate of one subgroup by the other unlike the case for D, we divide one by another.

The NMR estimate for the reference subgroup, *yref,* and the national average of neonatal mortality rate was used to compute PAR. For ordered dimensions the most-advantaged sub-group describes y_ref_, which in our case are the secondary school and above subgroups for education and richest sub-group for economic status and for binary dimensions like sex and residence, y_ref_ refers to the subgroup which has the lowest estimate, which in our case are female and urban residence. For non-ordered dimensions like subnational region, y_ref_ points out the subgroup or region with the lowest estimate, PAF were computed by dividing the PAR by the national average μ and multiplying the fraction by 100 (PAF = [PAR / μ] * 100). PAF is zero if no further improvement can be achieved, i.e. if all subgroups have reached the same level of health as the reference group (20). The change in NMR over time was assessed in reference to the 95% Confidence Intervals (CIs) of the different survey years. Whereas an absence of overlapped CIs portrays statistically significant difference between the two CIs, an overlap of CIs is an evidence of absence of inequality.

### Ethical consideration

The analyses were performed using publicly available DHS data. Institutions involved in the commissioning, funding and management of the surveys are responsible for ethical procedures in the surveys. To ensure that the survey protocols comply with the U.S. Department of Health and Human Services regulations for the protection of human subjects, all DHS obtain their approval from ICF international in addition to an Institutional Review Board (IRB) in the respective country.

## Results

In this study, a total of 40, 580 total live births were included based on mothers’ participation in the 2010 and 2016 surveys. Out of this sample, 20,090 (49.5%) were female newborns and 37,115 (91.4%) were rural residents. About 8813 births (21.7%) were born to women in the poorest wealth quintile; 21,201 (52.2%) were born to women with no formal education, followed by 16,303 (40.1%) babies born to women with a primary level of education.

The NMR (deaths per 1000 live births) in Burundi in 2010 and 2016 were 36.7 and 25.0 deaths per 1000 live births, respectively. NMR was nearly similar across economic subgroups in 2010, with the richer wealth quintile having slightly higher NMR than others. In 2016, NMR was lowest in the poorer wealth quintile which was approximately 19 neonatal deaths per 1000 live births (95% CI; 14.27, 23.96). Except in the poorest and richest groups, the NMR decreased in the other wealth categories between 2010 and 2016. For instance, the NMR was 36.93 (95% CI; 29.6, 45.93) and 33.85 (95% CI; 28.12, 40.71) among the poorest subgroup in 2010 and 2016 respectively, indicating no significant decrement in NMR over the last 6 years. In 2010, NMR was lower among the secondary education group with a rate of 18 neonatal deaths per 1000 live births (95% CI; 9.99, 32.04). However, no significant difference in NMR was seen in 2016 across education subgroups. While the secondary education subgroup had almost a constant NMR pattern over time, the NMR decreased in the other two groups (Table 1).

**Table 1:** Neonatal mortality rate (deaths per 1000 live births) disaggregated across the socio-economic, geographic and sex subgroups in Burundi from 2010 to 2016

Inequality dimension	Subgroups		2010		2016	
			Estimate (95% CI)	Pop^**n**^	Estimate (95% CI)	Pop^**n**^
**Economic status**	Poorest		36.93 (29.6, 45.93)	2948	33.85 (28.12, 40.71)	5865
	Poorer		35.54 (28.29, 44.57)	3118	18.50 (14.27, 23.96)	5495
	Middle		38.37 (28.82, 50.91)	2985	24.99 (19.68, 31.67)	5339
	Richer		42.11 (33.60, 52.67)	2852	25.40 (20.40, 31.59)	5121
	Richest		29.70 (22.08, 39.83)	2504	21.04 (15.16, 29.14)	4350
**Education**	No education		39.64 (34.69, 45.27)	7916	26.88 (23.26, 31.04)	13,285
	Primary school		35.17 (28.62, 43.15)	5712	23.09 (19.67, 27.10)	10,591
	Secondary school		17.95 (9.99, 32.04)	780	23.37 (15.17, 35.86)	2295
**Place of residence**	Rural		37.91 (33.86, 42.42)	13,281	24.99 (22.31, 27.98)	23,834
	Urban		22.46 (16.73, 30.10)	1128	25.56 (16.43, 39.57)	2337
**Sex**	Female		31.72 (27.05, 37.17)	7147	23.29 (20.01, 27.09)	12,943
	Male		41.59 (36.05, 47.95)	7262	26.75 (23.15, 30.89)	13,228
**Subnational region**	**Regions in 2010**	**Regions in 2016**				
	01 Bujumbura	01 Bubanza	19.75 (11.99, 32.36)	676	25.82 (13.85, 47.61)	1444
	02 North	02 Bujumbura rural	37.99 (31.35, 45.97)	4337	17.50 (12.09, 25.27)	485
	03 Centre-east	03 Bururi	33.77 (28.02, 40.65)	3537	19.59 (11.75, 32.49)	744
	04 West	04 Cankuzo	44.27 (35.37, 55.28)	2865	36.45 (25.75, 51.37)	828
	05 South	05 Cibitoke	34.85 (25.84, 46.86)	2992	26.89 (18.64, 38.66)	1606
	NA	06 Gitega	NA	NA	28.72 (19.70, 41.69)	2122
	NA	07 Karusi	NA	NA	16.31 (11.05, 24.00)	1439
	NA	08 Kayanza	NA	NA	21.75 (13.76, 34.24)	1656
	NA	09 Kirundo	NA	NA	37.46 (29.40, 47.62)	2093
	NA	10 Makamba	NA	NA	14.60 (8.78, 24.20)	1550
	NA	11 Muramvya	NA	NA	25.78 (16.85, 39.26)	900
	NA	12 Muyinga	NA	NA	37.97 (26.80, 53.55)	2247
	NA	13 Mwaro	NA	NA	16.47 (9.96, 27.11)	747
	NA	14 Ngozi	NA	NA	27.48 (18.12, 41.47)	2025
	NA	15 Rutana	NA	NA	21.04 (13.45, 32.74)	1102
	NA	16 Ruyigi	NA	NA	12.43 (7.85, 19.62)	1415
	NA	17 Bujumbura mairie	NA	NA	29.99 (15.22, 58.24)	1235
	NA	18 Rumonge	NA	NA	18.45 (10.59, 31.97)	1527
National			36.7		25.0	40,580

*NA* Not applicable for 2010 survey

### Extent and time trends of socio-economic and area-based inequality

Table 2 shows NMR inequality with respect to wealth, education, residence, sex, and sub-national region dimensions for both 2010 and 2016. We organized the main findings into socio-economic, area-based and sex-related inequality.

**Table 2:** Magnitude and trends of inequalities in neonatal mortality rate (deaths per 1000 live births) in Burundi from 2010 to 2016

Dimension		2010	2016
	Measure	%(95%CI)	%(95%CI)
Economic status	D	7.23 (−4.68, 19.14)	12.81 (3.51, 22.10)
	PAF (%)	−19.07(−23.55, −14.58)	−15.95(−20.42, −11.48)
	PAR	−6.99 (−8.64, −5.35)	−3.99 (−5.11, −2.87)
	R	1.24 (0.78, 1.69)	1.60 (1.00, 2.21)
Education	D	21.69 (9.98, 33.40)	3.50 (−7.26, 14.27)
	PAF(%)	−51.06(− 58.30, −43.82)	−6.64 (− 13.27, −0.02)
	PAR	− 18.74 (− 21.4, − 16.08)	− 1.66 (− 3.32, 0.00)
	R	2.20 (0.88, 3.52)	1.14 (0.62, 1.67)
Place of residence	D	15.44 (7.59, 23.29)	−0.57 (− 12.15, 11.00)
	PAF(%)	−38.78(−45.24, − 32.32)	0 (− 6.73, 6.73)
	PAR	− 14.23 (− 16.60, − 11.86)	0 (− 1.68, 1.68)
	R	1.68 (1.15, 2.21)	0.97 (0.53, 1.42)
Sex	D	9.87 (2.09, 17.65)	3.45 (−1.76, 8.68)
	PAF(%)	−13.55(− 15.72, − 11.39)	−6.97 (−9.09, −4.86)
	PAR	−4.97 (−5.76, − 4.18)	−1.74 (−2.27, −1.21)
	R	1.31 (1.03, 1.59)	1.14 (0.90, 1.38)
Region	D	24.51 (10.60, 38.43)	25.54 (11.22, 39.85)
	PAF(%)	−46.18(−54.26, −38.1)	− 50.34 (− 57.12, − 43.56)
	PAR	−16.94 (−19.91, − 13.98)	−12.60 (−14.30, −10.90)
	R	2.24 (1.02, 3.46)	3.05 (1.30, 4.80)

NB: The national average NMR was 36.7 deaths per 1000 live births in 2010 (using births in the 5 preceding years) and 25.0 in 2016.; For PAR and PAF the reference are the most advantaged subgroups (least burdened) (richest, secondary school and above, urban, female, region with lowest NMR such as Bujumbura in 2010 and Ruyigi in 2016); For Difference and Ratio the reference was the subgroup with highest value (highest NMR)

*D* Difference; *PAR* Population Attributable Risk; *PAF* Population Attributable Fraction; *R* Ratio

### Socioeconomic inequality

The socio-economic inequality consisted of wealth-based and educational status inequality. Wealth based inequality in NMR was observed in Burundi by complex measures (PAF, PAR) in 2010 (PAF = − 19.07, 95% CI; − 23.55, − 14.58, PAR = − 6.99, 95% CI; − 8.64, − 5.35) and 2016 (PAF = − 15.95, 95% CI; − 20.42, − 11.48, PAR = − 3.99, 95% CI; − 5.11, − 2.87). Additionally, the D measure also indicates presence of economic inequality in 2016. The relative inequality was higher than the absolute inequality in both years, with the absolute inequality decreasing over time. Educational status inequality was observed in 2010 by all the measures except R (R = 2.20, 95% CI; 0.88, 3.52). In 2016, only relative inequality existed based on PAF (PAF = − 6.64, 95% CI; − 13.27, − 0.02). In terms of time trend, the inequality decreased.

### Area based inequality

Both absolute and relative place of residence inequalities in NMR were observed in 2010 by all the measures. However, all measures did not indicate existence of inequality in 2016. For instance, the Dmeasure showed (15.44, 95% CI; 7.59, 23.29) and (− 0.57, 95% CI; − 12.15, 11.00) in 2010 and 2016 respectively, indicating significant absolute urban-rural disparities in 2010 but not in 2016. Similarly, the PAF measure indicated (− 38.78, 95% CI; − 45.24, − 32.32) and (0, 95% CI; − 6.73, 6.73) in 2010 and 2016 respectively, signifying substantial urban-rural relative disparities in 2010 but not in 2016.

Another main finding from the current study is the presence of subnational region inequality in NMR in 2010 and 2016 by all measures. The pattern of region-based inequality in NMR was roughly constant overtime. For instance, the D measure (24.51, 95% CI; 10.60, 38.43 and 25.54, 95% CI; 11.22, 39.85) in 2010 and 2016 respectively, indicating significant absolute regional inequality with overtime constant pattern. Again, the R measure (2.24, 95% CI; 1.02, 3.46 and 3.05, 95% CI; 1.30, 4.80) in 2010 and 2016, indicating substantial relative regional disparities in NMR with constant pattern.

### Sex related inequality

Sex inequality in NMR was observed in 2010 by all measures and in 2016 by complex measures only (PAF = − 6.97, 95% CI; − 9.09, − 4.86, PAR = − 1.74, 95% CI; − 2.27, − 1.21), with both absolute and relative inequalities decreasing over time.

## Discussion

In this study, we examined the over-time socioeconomic, sex-related and geographical disparities in NMR in Burundi. Our findings highlighted significant inequalities in NMR between the rich and the poor, educated and non-educated groups, male and female neonates, urban and rural, and between regions, with varying over time trends. We noted statistically important wealth-driven inequality in NMR both in 2010 and 2016 that favors the rich. While the pattern of absolute wealth-driven inequality showed decreasing trend from 2010 to 2016 by PAR measure, the relative wealth-based disparity remained constant during the same time period. The finding that NMR is highly prevalent among the poor is consistent with previous studies in many countries [22–24].

The reason for a higher NMR among the poorest subpopulation might be due to difference in the household’s coping capacities in the country’s crisis which exacerbated the nation’s existing food security and malnutrition challenges caused by the disruption of food supplies [17, 25–27]. Political instability and insecurity in Burundi since mid-2015, coupled with natural disasters, have resulted in falling agricultural production and economic contraction [17, 26]. This in turn has resulted in a food insecurity crisis among vulnerable households [17, 26]. The ongoing political tension disrupted crop production, as many farmers had to abandon their land due to insecurity [17, 25, 26].

Our study also reveals substantial absolute and relative education-based inequality in NMR in 2010 and a far less severe relative and absolute inequalities in the 2016 surveys. These results suggest a higher number of neonatal deaths among newborns of uneducated mothers compared to educated mothers. If the observed educational disparities were prevented, the country could have reduced the 2010 and 2016 NMR, based on the point estimates of the PAF measure, approximately by 51 and 7% respectively. It’s worth noting that both the relative disparities were significantly reduced from 2010 to 2016 and the absolute education-related inequality could not be detected in 2016 altogether, a finding that might signal the country’s commitment in providing good health care services for the entire population including the uneducated. Consistent with a previous study [28], our finding also shows lower NMR among newborns of educated mothers. This could be due to better birth spacing, better awareness and utilization of prenatal care and health services among educated mothers [29, 30]. Participation in higher education allows a woman to gain a better occupation and hence a higher income/wealth level [31, 32]. It also influences the attitudes of mothers towards traditional norms and beliefs, which have influence on the neonatal, infant and child survival [33]. Education can be anticipated to be correlated with an increased appreciation of illnesses, signs, and symptoms, and accessibility of services and it’s a good proxy of socioeconomic position [34, 35].

We also found a considerable pro-urban inequality in NMR in 2010. The Neonatal mortality rate in 2010 among rural residents was higher by more than 15 neonatal deaths per 1000 live births, compared to their urban counterparts. Similarly, as detected by the R measure, NMR among rural residents was 1.68 times higher than in urban residents. In 2010, NMR would have been reduced by 39%, based on the point estimates, if Burundi had put in place relevant interventions to reduce relative inequalities. This higher NMR among rural residents is consistent with a previous study in Cambodia [28]. The reason for high mortality in rural setting might be related to congenital malformations, which are more common in rural residents [35]. Evidence shows that congenital malformation in the neonatal and post neonatal period is associated with higher risk of mortality in rural areas [35]. The causes of most congenital malformations are not yet identified; nevertheless, it is possible that their risk factors include the limited access to prenatal care [36] occupational exposure to pesticides [37] and low socio-economic status [38].

Our results also highlight considerable absolute and relative sex-based inequality both in 2010 and 2016 with higher concentration of NMR among male newborns. Effective strategies would have helped reduce NMR both in 2010 and 2016, NMR by nearly 14 and 7% respectively with regard to relative inequalities, and by 5 and 2 neonatal deaths per 1000 live births for absolute sex-based inequalities respectively, based on the point estimates, during the same periods. Male infants seem to be more vulnerable to mortality and morbidity, including intrauterine growth restriction, respiratory insufficiency or prematurity [39]. The underlying mechanisms contributing to the observed male disadvantage have not been elucidated. At present, some hypotheses have been proposed regarding the role of gender-associated genetic and endocrine differences in the determination of neonatal mortality or morbidity. Authors of previous studies have speculated that the male disadvantage might be caused by hormonal environment differences and absolute sex-based inequalities [40].

Substantial absolute and relative subnational regional inequalities in NMR are found in both 2010 and 2016, favoring newborns from certain regions than others. For instance, if the absolute regional inequality were avoided, the 2010 and 2016 country’s NMR estimate would be reduced by nearly 17 and 13 neonatal deaths per 1000 live births respectively. Evidence suggests the possible reasons for this disparities are related to difference in quality of midwifery, obstetric, and pediatric care available [7]. Additionally, differences in the number of health facilities and health personnel, easiness or difficulty in accessibility to health services, and number of private health facilities within the regions can largely create disparities in health service qualities including pregnant women across regions [7, 41]. A major difficulty underlying the low quality in the delivery of healthcare services in Burundi is the shortage of qualified human resources [7], difference in distribution of health human resources. Efforts to redistribute medical staff more equitably according to the Ministry’s norms have been hampered by the lack of incentives for medical staff to move to remote areas, as well as the absence of accountability and sanction mechanisms governing the distribution of medical staff. Accessibility of facility might create a good opportunity for utilization of health services [42, 43], which can help address newborn deaths through early detection and management of pregnancy problems [44].

It has been shown that health care inequality monitoring requires disaggregation of a health care indicator by appropriate and relevant social inequality dimensions [21]. Measures of inequality have the potential to reveal why reducing unfavorable health care indicator disparities between subgroups is important in terms of reducing the aggregate national figure of the same indicator. From our analysis, for example, if there were no education related disparity in NMR in 2010, the national NMR would have been reduced by more than 50%, that is, the NMR in 2010 would be 18 deaths per 1000 live birth instead of 37 deaths per 1000 live births (the 2010 NMR for Burundi). Disparities in NMR are thus considered as barriers in decreasing national level NMR and this in turn, affects the prospect of attaining the 2030 SDG of the NMR [4]. Evidence shows that reducing inequality between different population groups would result in gains in health status [45].

### Strengths and limitations

The study has numerous strengths. First, the use of several measures of inequality contributed to the worth of evidence contained in this paper. The weakness of each method is better supplemented by the strengths of other methods. Also, using both relative and absolute inequality measures in the same study has the potential to help examine the magnitude and trend of inequality from a number of dimensions and viewpoints. Secondly, the study presented the inequality findings for each subgroup of the equity stratifiers, and this can assist the government to identify where and how to focus their efforts towards realization of the equity-oriented SDG in relation to neonatal health. Finally, the study used a high-quality data available through the WHO health equity monitor database which strengthens the quality of the conclusions drawn from the study.

Our study should be viewed by considering a number of limitations. The study presented only the population level NMR inequality at the national level, and further small studies at district and kebele level may be required to see whether the same disparity remains. Our study focused on description of the nature of NMR inequality in light of the recommended dimensions of health inequality. We recommend the conduct of decomposition analysis to explore factors that could explain the disparities in NMR across various dimensions of inequality observed in this study. To fully account for the overtime variations in the distribution of wealth and education in each region, we recommend further research on wealth and education-based inequalities studies in each region and over time. Moreover, the gold standard source of data on NMR is the vital registration system. However, many LMICsincluding Burundi have either no or incomplete vital registration system, and therefore population based surveys including DHS remain the best data source of NMR. Although they tend to suffer problems such as omission, heaping and misclassification of deaths, DHSs continue to be the standard source of data for measuring NMR and are likely to show the actual burden of NMR than facility based studies since the substantial amount of births and deaths in SSA occur outside of the health facilities. Moreover, in 2010, in the case of place of residence, PAF is missing if the estimate for the reference subgroup or if the population share for at least one subgroup is missing. This also affects the comparison with 2016 as the regions compared are not the same. Finally, the change in NMR estimate in the country over time might reflect the improvement in the reporting of the neonatal deaths between time periods. However, the DHS methodologies are essentially similar between the two rounds and both surveys used the same methodology to estimate NMR.

## Conclusion

This paper presents evidence on NMR disparity in Burundi and found that enormous survival advantages remains in neonates born into well-to-do families. Also, lower NMR was found in neonates born to more educated mothers, urban residents, and certain regions in the country. We also showed that male neonates had higher chances of dying than females, creating pro-female scenario of NMR in both 2010 and 2016. While residence and absolute education-related NMR disparities had gone between 2010 and 2016 and absolute wealth-driven inequality and relative economic inequality dramatically decreased, subnational region and relative economic disparities persisted between 2010 and 2016. The government could consider revising current interventions to develop strategies that target more affected subgroups in order to eliminate the avoidable disparity.

## Data Availability

The datasets generated and/or analyzed during the current study are available in the WHO’s HEAT version 3.1 [https://www.who.int/gho/health_equity/assessment_toolkit/en/].

## Abbreviations

D: Difference
NMR: Neonatal Mortality rate
BDHS: Burundi Demographic and health Survey
EA: Enumeration Area
HEAT: Health Equity Assessment Toolkit
PAR: Population Attributable Risk
PAF: Population Attributable Fraction
PPS: Probability Proportional to Size
R: Ratio
WHO: World Health Organization
SDG: Sustainable Development Goal
